# Characteristics and Patterns of Metastatic Disease from Chordoma

**DOI:** 10.1155/2015/517657

**Published:** 2015-12-30

**Authors:** Victoria A. Young, Kevin M. Curtis, H. Thomas Temple, Frank J. Eismont, Thomas F. DeLaney, Francis J. Hornicek

**Affiliations:** ^1^Department of Orthopaedics, University of Miami Miller School of Medicine, P.O. Box 016960 Miami, FL 33101, USA; ^2^University of Miami Tissue Bank, 1951 NW 7th Avenue, Suite 200, Miami, FL 33136, USA; ^3^Department of Biochemistry & Molecular Biology, University of Miami Miller School of Medicine, P.O. Box 016960 Miami, FL 33101, USA; ^4^Geriatric Research, Education, and Clinical Center and Research Service, Bruce W. Carter Veterans Affairs Medical Center, Miami, FL 33125, USA; ^5^Department of Radiation Oncology, Massachusetts General Hospital, Harvard Medical School, Francis H. Burr Proton Therapy Center (FHBPTC), 55 Fruit Street, Boston, MA 02114, USA; ^6^Department of Orthopedic Surgery, Massachusetts General Hospital, Harvard Medical School, Orthopaedic Associates, 55 Fruit Street Yawkey 3B, Boston, MA 02114, USA

## Abstract

Chordoma is a rare, slow-growing malignant tumor arising from notochordal remnants. A retrospective review of patient records at two major referral centers was undertaken to assess the incidence, location, and prognostic factors of metastatic disease from chordoma. 219 patients with chordoma (1962–2009) were identified. 39 patients (17.8%) developed metastatic disease, most frequently to lung (>50%). Median survival from the time of initial diagnosis was 130.4 months for patients who developed metastatic disease and 159.3 months for those who did not (*P* = 0.05). Metastatic disease was most common in the youngest patients (*P* = 0.07), and it was 2.5 times more frequent among patients with local recurrence (26.3%) than in those without (10.8%) (*P* = 0.003). Patient survival with metastatic disease was highly variable, and it was dependent on both the location of the tumor primary and the site of metastasis. Metastasis to distal bone was the most rapid to develop and had the worst prognosis.

## 1. Introduction

Chordoma is a rare, slow-growing, malignant tumor arising from notochordal remnants. Chordoma accounts for 1–4% of primary bone tumors [1, 2] and has an estimated incidence of 8/10,000,000/year [3]. Chordoma occurs primarily in the fifth and sixth decades of life and more frequently affects males (2 : 1), yet demographics can vary based on site of presentation [2–4]. Due to a slow growth rate and nonspecific presentation, diagnosis is delayed 1.5 years on average [5], leading to more advanced disease by the time treatment is initiated [2]. Despite its slow progression, patients with chordoma have a poor prognosis, likely due to tumor location and its propensity toward local recurrence (68%) [5–9], resulting in diminished 5-year (67%) and 10-year (40%) survival [3].

Since its description 150 years ago there has been relatively sparse clinical data collected on chordoma. The few studies describing metastatic disease in chordoma report an estimated frequency of metastasis ranging from 3 to 48% [4] and suggest that the presence of metastasis increases the risk of tumor-related death [6]. Metastasis is relatively uncommon, with lung representing the most common site. Metastases to liver, bone, lymph nodes, skin, subcutaneous tissue, muscle, peritoneum, heart, pleura, spleen, kidney, bladder, pancreas, and brain have also been described [6, 9–11]. Factors that may be predictive of metastasis include local recurrence, large tumor size, inadequate surgical margins, necrosis, long clinical duration, and exposure to high dose radiation [4, 9, 11]. However, a recent study reported no significant difference in likelihood of metastasis based on age, gender, tumor location, or radiation therapy [9]. Earlier studies suggest a relationship between primary tumor location and metastatic disease risk [4, 12, 13], though the strength of this correlation is uncertain as these reports contain conflicting data and relatively few patients [6]. To better understand patterns of metastatic disease from chordoma, we conducted a retrospective review of 219 patients with primary chordoma, 39 of whom developed metastatic disease.

## 2. Methods

### 2.1. Patient Data Collection

A retrospective review of all cases of chordoma diagnosed from November 1962 to October 2009 at two major US referral centers, Mass General Hospital, Boston, MA, and University of Miami, Miami, FL, was undertaken. Patient characteristics were recorded and charts, radiographs, and histological slides were reviewed for sites of primary and metastatic lesions. Bone scans, MRI, chest radiographs, and/or CT scans were also reviewed. The following data were obtained or defined: patient age, gender, site of primary tumor, local recurrence, metastasis, site of primary metastasis, presence of multiple metastasis, site of secondary metastasis, and survival. Due to the rare incidence of chordoma and lack of published large scale retrospective studies, we focused only on the prognostic factors of the disease and did not include the effects of treatment or cause of patient death.

**Figure 1:**
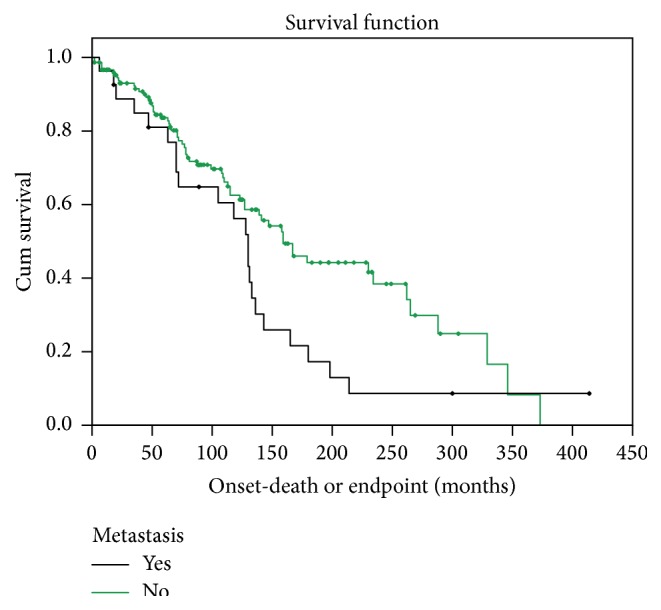
Survival distribution stratified by presence of metastatic disease. Diamonds represent censored data points.

**Figure 2:**
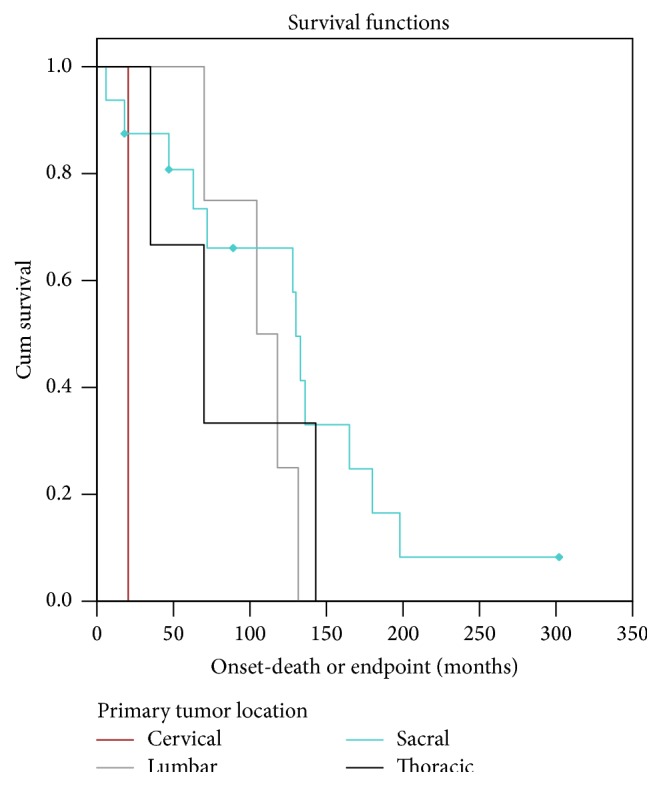
Survival distribution stratified by primary tumor location. Diamonds represent censored data points.

Frequencies and descriptive statistics were obtained for analysis; categorical variables were examined using chi-square and Fisher's exact tests, where appropriate. Kaplan-Meier survival curves with log rank analysis were performed for survival, time from onset to metastasis, and time from metastasis to death. Survival was defined as time from diagnosis to date of death. Patients surviving beyond the study cutoff date were censored. There was sufficient data to analyse 219 patients, yet only 177 cases of primary chordoma could be used for survival analysis (see Figure 1 and Table 2(a)). Excluded from the analysis were individuals known to be deceased, but for which no date of death could be defined, and those lost to follow-up whose status (living or deceased) was unknown. Excluded from analyses involving primary tumor location were individuals with unknown, multiple, or atypical sites of presentation (see Figure 2 and Table 5(a)). Censored observations and data points are presented as vertical hashmarks in the survival curves. This study was approved by the institutional review boards of the respective institutions: UM/JMH IRB 20100483 and MGH IRB 2003-P-000987/5.

## 3. Results

### 3.1. Patient Population and Disease Presentation

219 patients with chordoma presented to the Orthopaedics Services at two major referral centers in a 46-year span (Table 1). There were 137 (63%) men and 81 (37%) women. Age at diagnosis was divided into quartiles, with most individuals in the third quartile (median age at diagnosis 57, range: 3–88 years). The most frequent site of presentation was sacral (60.7%). A few primary tumors were documented in the clivus/skull base (1.8%), and the remaining were observed in the mobile spine (34.7%), which is further subdivided into lumbar (17.4%), cervical (10.0%), and thoracic (7.3%).

Chordoma location at initial presentation trended toward a dependence on patient age (*P* = 0.06). The youngest patients (Q1: 3–24 yrs old) tended to have atypical primary tumor locations, with chordoma appearing in the cervical spine (50%, *n* = 4) more frequently than the sacrum (37.5%, *n* = 3) and clivus (12.5%, *n* = 1). The remaining age quartiles (Q2–Q4: >25 yrs old) were more likely to have involvement in the typical/classical sacrococcygeal region (62.5–64.4%) than in the lumbar (12.5–21.4%), cervical (6.7–14.6%), thoracic spine (7.1–8.9%), and clivus (0.9–2.2%). Patients with youngest age of presentation (Q1) were more likely to be female (56% F : 44% M) as compared to older quartiles Q2–Q4 where males predominated (Q2–Q4 = 27–38% F : 60–73% M). Additionally, patients with primary tumors of the cervical spine were more likely to be female as well (54.4% F : 45.5% M). Clival chordomas were found to occur equally among the genders, with sacral, thoracic, and lumbar chordoma more frequently occurring in males (64.4–68.8% M : 31.3–35.6% F).

### 3.2. Overall Survival

Median overall survival was 140.5 months (95% CI 115.7–165.3) (Table 2(c)). The duration of survival differed based on site of presentation; individuals with primary tumors of the cervical and thoracic spine had the shortest median survival at 74.7 and 76.8 months, respectively. Those with primary tumors of the lumbar spine had median survival of 126.7 months, while those with primary tumors of the sacrum had the longest median survival at 159.3 months.

While median survival did not differ based on presence of local disease recurrence alone, median survival among patients with metastatic disease (Table 2(a)) was significantly (*P* = 0.05) (Table 2(b)) lower (130.4 months [95% CI 111.4–149.5]) than patients without metastatic disease (159.3 months [95% CI 123.9–194.6]) (Figure 1, Table 2(c)).

### 3.3. Metastasis

Of the 39 (17.8%) patients who developed metastatic disease, lung was most frequently involved (53.8%). Other sites of primary metastasis include distal bone (20.5%), soft tissue (15.4%), and liver (7.7%) (Table 3). Metastatic disease was most frequent among the youngest patients (Q1 44%, Q2 16%, Q3 20%, and Q4 9%) (*P* = 0.07) and 2.5 times more frequent among patients with local recurrence (26.3%) than without (10.8%) (*P* = 0.003). Tumors presenting in the cervical spine rarely became metastatic (1 [4.5%] of 22), whereas tumors in the remainder of the mobile spine demonstrated metastatic rates ranging from 17 to 32% (Table 4). Throughout the course of their illness, 99 patients (45.2%) developed locally recurrent disease (Table 1), with lumbar chordoma being the most likely to recur. If disease was first locally recurrent, these lesions took longer to metastasize (67.2 months versus 20.5 months) (*P* ≤ 0.001) and progressed more slowly from the diagnosis of metastasis to death (22.1 months) than disease that was directly metastatic (4.5 months).

The time from onset to metastasis was found to differ significantly, depending on the site of presentation (*P* = 0.013). The shortest duration from onset to metastasis was found among patients with primary tumors located in the cervical spine (16.0 months). Time to metastasis of primary tumors located in the thoracic spine, lumbar spine, and sacrum was 22.0 months, 49.2 months, and 58.3 months, respectively. The longest time to metastasis was 120.1 months, which was observed in a patient with a primary clival chordoma.

### 3.4. Survival among Patients with Metastatic Disease

Among patients with metastasis (Table 5(a)), survival differed based on primary tumor location (*P* = 0.05) (Table 5(b)). Patients with metastatic tumors originating in the cervical spine had the shortest median survival (20.4 months, *n* = 1), followed by tumors of the thoracic spine (70.1 months, *n* = 3), lumbar spine (104.9 months, *n* = 4), and sacrum (130.4 months, *n* = 16) (Figure 2, Table 5(c)).

**Figure 3:**
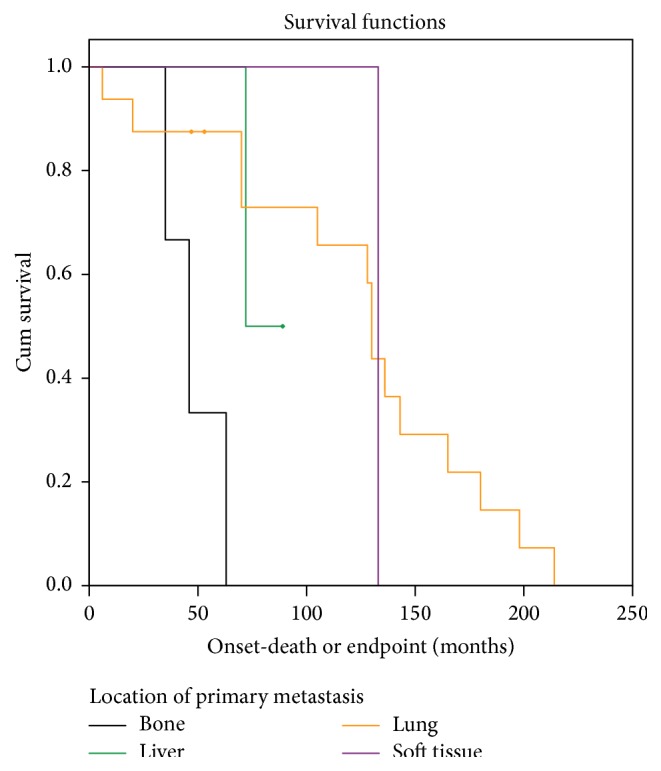
Survival distribution among patients with metastasis stratified by location of metastatic disease. Diamonds represent censored data points.

Survival differed significantly based on site of metastasis (*P* = 0.01) (Tables 6(a) and 6(b)). Tumors that first metastasized to bone had the shortest median survival at 46 months, followed by liver at 72 months (Table 6(c)). Sites of primary metastasis with the longest median survival were lung and soft tissue, at 130 months and 132 months, respectively (Figure 3, Table 6(c)).

## 4. Discussion

### 4.1. Disease Presentation

In agreement with previous studies, individuals presenting with the typical sacrococcygeal chordoma tended to be males in the 6th and 7th decades of life, with a relatively good prognosis. Our data demonstrate, also in agreement with previous studies, that patients over 25 years of age were more likely to be male and to present with chordoma in the sacral, thoracic, or lumbar spine [12, 14]. The literature also describes a younger, atypical population of chordoma patients with cranial primaries [3, 15], but our data suggest that this atypical, young female predominant group (under 25 yo.) has a predisposition toward aggressive chordomas of the cervical spine. And although this atypical group of patients represents less than 5% of the chordoma population, cervical chordoma presentation has the lowest survival of 74.7 months. Thus, in concordance with prior study, our data reproduce two distinct groups: (1) an atypical group of young female patients who predominantly present with a more aggressive form of cervical chordoma, and (2) a more common, typical group composed predominantly of older male patients with sacral, thoracic, and lumbar tumors. It is unclear from our data whether the relatively poor prognosis of the atypical female cervical chordomas is secondary to more aggressive inherent tumor properties or to the anatomical constraints imposed on surgical intervention in the cervical spine.

### 4.2. Predicting Metastasis

In concordance with prior literature, nearly half of the individuals in the present study had local recurrence during the course of their disease. As in the literature, we found a significant correlation between the presence of local recurrence and the subsequent development of metastasis [4, 6, 9]. Interestingly, in contrast to this general rule, while lumbar chordoma was often recurrent, there was a relative lack of metastatic disease originating from this site. The strong correlation between local recurrence and subsequent metastasis reinforces the importance of local tumor control, as our data suggest that chordomas may acquire metastatic potential with uncontrolled growth at the primary site.

### 4.3. Patterns of Metastatic Disease and Prognosis

There is a paucity of data in the chordoma literature describing the expected time to metastasis. One study addressed this issue, reporting times to metastasis ranging from 0.2 to 13.3 years [6]. In our large cohort, we found the median time from initial diagnosis to metastasis to be 4.8 years (58.3 mo), with most individuals developing metastasis between 2.9 and 6.7 years. After metastatic disease is diagnosed, our data show survival to be highly variable, dependent on both the location of the tumor primary and the site of metastasis, with a median of 1.7 years between the diagnosis of metastatic disease and patient death, similar to one study by Bergh et. al. [6]. Metastasis to distal bone was the most rapid to develop and had the worst prognosis.

Although initially contested, recent literature identifies lung to be the most common site for chordoma metastasis [9]. In our data, lung metastases account for more than 50% of all metastatic disease, making lung metastases approximately three times more common than bone and soft tissue, which are the second and third most frequent sites of metastatic disease. And interestingly, metastatic disease to bone and soft tissue almost always arises from locally recurrent chordoma. In contrast, metastasis to the lung was associated with prior local recurrence in only half of the cases.

## 5. Conclusion

Our large retrospective study pooled data from two major cancer centers to characterize the incidence, location, and prognostic factors of metastatic disease in patients with primary chordoma. Lung is the favored site for metastasis (>50%). Metastatic disease most commonly occurred in the youngest patients (<25 years old) (*P* = 0.07), and it was 2.5 times more frequent among patients with local recurrence (26.3%) than in those without (10.8%) (*P* = 0.003). Patient survival with metastatic disease was highly variable, and it was dependent on both the location of the tumor primary chordoma and the site of metastasis. But overall, metastatic disease is a poor prognosticator. The median survival from the time of initial diagnosis is 130.4 months for patients who developed metastatic disease and 159.3 months for those who did not (*P* = 0.05). Metastasis to distal bone was the most rapid to develop and had the worst prognosis.

## Figures and Tables

**Table 1:** 

Patient characteristics		*n* (%)
Total number		219

Gender	Male	137 (63)
	Female	81 (37)

Age (years)	Median	57
	Range	3–88

Age quartiles	Q1: (3–24 yrs)	9 (4.1)
	Q2: (25–46 yrs)	49 (22.4)
	Q3: (47–68 yrs)	116 (53)
	Q4: (69–88 yrs)	45 (20.5)

Presenting site	Clivus	4 (1.8)
	Cervical spine	22 (10.0)
	Lumbar spine	38 (17.4)
	Thoracic spine	16 (7.3)
	Sacrum	133 (60.7)
	Multiple	4 (1.8)

Extension of disease	Primary only	107 (47.4)
	Recurrent (−) metastasis	73
	Recurrent (+) metastasis	26
	Total locally residual/recurrent disease	99 (45.2)
	Metastasis (+) local recurrence	26
	Metastasis (−) local recurrence	13
	Total metastatic	39 (17.8%)

**Table:** (a) Case processing summary of patients included in analysis

Metastasis	Total *N*	*N* of events	Censored	
			*N*	Percent
No	150	63	87	58.0%
Yes	27	22	5	18.5%

Overall	177	85	92	52.0%

**Table:** (b) Overall statistical comparison and test of equality of survival distributions

	Chi-Square	df	Sig.
Log rank (Mantel-Cox)	3.682	1	**0.055**

**Table:** (c) Means and medians for survival time. Estimation is limited to the largest survival time if it is censored

Metastasis	Mean					Median			
			95% Conf. interval					95% Conf. interval	
	Estimate	Std. error	Lower bound	Upper bound		Estimate	Std. error	Lower bound	Upper bound
No	188.5	13.9	161.3	215.6		**159.3**	18.1	123.9	194.6
Yes	134.2	20.6	93.8	174.6		**130.4**	9.7	111.4	149.5

Overall	177.8	12.5	153.3	202.2		140.5	12.7	115.7	165.3

**Table 3:** Primary metastasis.

Primary metastatic site	Total (*n*)	%
Lung	21	53.8%
Liver	3	7.7%
Bone	6	15.4%
Sternum	2	5.1%
Soft tissue	6	15.4%
Site not listed	1	2.6%
**Total**	**39**	**17.8%**

**Table 4:** Patterns of primary metastasis.

Primary tumor site	Total (*n*)	Metastatic (*n*)	%	Site of primary metastasis
Clivus	4	1	25%	Soft tissue [calf]

Cervical spine	22	1	4.5%	Lung

Lumbar spine	38	7	18.4%	Sternum (2)
				Lung (3)
				Soft tissue [1: abdomen, 1: groin and psoas muscle]

Sacrum	133	23	17.3%	Lung (13)
				Liver (3)
				Bone [1: lumbar spine, 3: thoracic spine, 1: multiple bone sites] (5)
				Soft tissue (2)

Thoracic spine	16	5	31.3%	Lung (3)
				Bone [trochanter] (1)
				Soft tissue [abdominal wall] (1)

Multiple primary sites	4	1		Lung

Posterior mediastinum	1	0		

Not documented	1	1		Lung

**Total**	**219**	**39**	**17.4%**	

**Table:** (a) Case processing summary of patients included in analysis

Primary tumor location	Total *N*	*N* of events	Censored	
			*N*	Percent
Cervical	1	1	0	0%
Lumbar	4	4	0	0%
Sacral	16	12	4	25%
Thoracic	3	3	0	0%

Overall	24	20	4	16.7%

**Table:** (b) Overall statistical comparison and test of equality of survival distributions

	Chi-Square	df	Sig.
Log rank (Mantel-Cox)	7.917	3	**0.048**

**Table:** (c) Means and medians for survival time. Estimation is limited to the largest survival time if it is censored

Primary tumor location	Mean					Median			
			95% Conf. interval					95% Conf. interval	
	Estimate	Std. error	Lower bound	Upper bound		Estimate	Std. error	Lower bound	Upper bound
Cervical	20.4	N/A	20.4	20.4		**20.4**	N/A	N/A	N/A
Lumbar	106	13.1	80.1	131.8		**104.9**	24.1	57.6	152.2
Sacral	127.9	20.7	87.4	168.4		**130.4**	3.8	123	137.9
Thoracic	82.6	31.8	20.4	144.9		**70.1**	28.7	13.9	126.3

Overall	111.7	14.6	83	140.5		128	18.4	92	164

**Table:** (a) Case processing summary of patients included in analysis

Location of primary metastasis	Total *N*	*N* of events	Censored	
			*N*	Percent
Bone	3	3	0	0%
Liver	2	1	1	50.0%
Lung	16	14	2	12.5%
Soft tissue	1	1	0	0%

Overall	22	19	3	13.6%

**Table:** (b) Overall statistical comparison and test of equality of survival distributions

	Chi-Square	df	Sig.
Log rank (Mantel-Cox)	11.260	3	**0.010**

**Table:** (c) Means and medians for survival time. Estimation is limited to the largest survival time if it is censored

Location of primary metastasis	Mean					Median			
			95% Conf. interval					95% Conf. interval	
	Estimate	Std. error	Lower bound	Upper bound		Estimate	Std. error	Lower bound	Upper bound
Bone	48	8.2	32	64		**46**	8.9	28.4	63.6
Liver	81	6	68.7	92.3		**72**	N/A	N/A	N/A
Lung	123.3	15.7	92.6	154.1		**130**	1.8	126.4	133.6
Soft tissue	133	0	133	133		**132**	N/A	N/A	N/A

Overall	111.6	13.1	86	137.4		128	16.8	97.1	163
